# Successful Proof of Concept of Family Planning and Immunization Integration in Liberia

**DOI:** 10.9745/GHSP-D-14-00156

**Published:** 2015-03-02

**Authors:** Chelsea M Cooper, Rebecca Fields, Corinne I Mazzeo, Nyapu Taylor, Anne Pfitzer, Mary Momolu, Cuallau Jabbeh-Howe

**Affiliations:** a Jhpiego, Baltimore, MD, USA; b John Snow, Inc, Arlington, VA, USA; c Independent Consultant, USA; d Jhpiego, Monrovia, Liberia; e Ministry of Health and Social Welfare, Monrovia, Liberia

## Abstract

Globally, unmet need for postpartum family planning remains high, while immunization services are among the most wide-reaching and equitable interventions. Given overlapping time frames, integrating these services provides an opportunity to leverage existing health visits to offer women more comprehensive services. From March through November 2012, Liberia's government, with support from the Maternal and Child Health Integrated Program (MCHIP), piloted an integrated family planning and immunization model at 10 health facilities in Bong and Lofa counties. Vaccinators provided mothers bringing infants for routine immunization with targeted family planning and immunization messages and same-day referrals to co-located family planning services. In February 2013, we compared service statistics for family planning and immunization during the pilot against the previous year's statistics. We also conducted in-depth interviews with service providers and other personnel and focus group discussions with clients. Results showed that referral acceptance across the facilities varied from 10% to 45% per month, on average. Over 80% of referral acceptors completed the family planning visit that day, of whom over 90% accepted a contraceptive method that day. The total number of new contraceptive users at participating facilities increased by 73% in Bong and by 90% in Lofa. Women referred from immunization who accepted family planning that day accounted for 44% and 34% of total new contraceptive users in Bong and Lofa, respectively. In Lofa, pilot sites administered 35% more Penta 1 and 21% more Penta 3 doses during the pilot period compared with the same period of the previous year, while Penta 1 and Penta 3 administration decreased in non-pilot facilities. In Bong, there was little difference in the number of Penta 1 and Penta 3 doses administered between pilot and non-pilot facilities. In both counties, Penta 1 to Penta 3 dropout rates increased at pilot sites but not in non-pilot facilities, possibly due to higher than average background dropout rates at pilot sites prior to the intervention in Lofa and the disproportionate effect of data from 1 large facility in Bong. The project provided considerable basic support to assess this proof of concept. However, results suggest that introducing a simple model that is minimally disruptive to existing immunization service delivery can facilitate integration. The model is currently being scaled-up to other counties in Liberia, which could potentially contribute to increased postpartum contraceptive uptake, leading to longer birth intervals and improved health outcomes for children and mothers.

## Abstract (precis)

Mobilizing vaccinators to provide mothers key family planning information and referrals to co-located, same-day family planning services was feasible in resource-limited areas of Liberia, leading to substantial increases in contraceptive use. Conversely, impact on immunization rates was less clear, but at a minimum there was no decrease in doses administered.

## BACKGROUND

Giving women access to family planning during the first year postpartum provides an opportunity to prevent unintended pregnancies and promote healthy birth spacing. Pregnancies spaced less than 18–24 months apart have been associated with an increased risk of preterm birth; low birth weight; fetal, neonatal, and infant death; childhood malnutrition and stunting; and adverse maternal health outcomes.^1^^,^^2^ More than 90% of women during their first year postpartum indicate a desire to delay the next pregnancy for at least 2 years, or to not get pregnant at all, yet there is substantial unmet need for family planning during this period.^3^

The Expanded Programme on Immunization (EPI) provides routine immunization to children in their first year of life, which corresponds to the extended postpartum period of their mothers. Routine immunization services are one of the most used and equitable health services: global coverage for the third dose of vaccine containing diphtheria, tetanus, and pertussis (DTP3) was estimated at 84% in 2013.^4^

Given that the time frames for EPI and postpartum family planning services overlap, integrating these services provides an opportunity to leverage existing contacts with the health system to offer women a more comprehensive package of services. Such integration of services has been recognized as a “promising” high-impact practice for improving access to family planning.^5^ Furthermore, the Global Vaccine Action Plan for 2011–2020 recognizes that strong immunization systems are an integral part of a well-functioning health system and states that immunization service delivery should continue to serve as a platform for providing other priority public health interventions.^6^

Integrating postpartum family planning and immunization services leverages existing contacts with the health system to offer women more comprehensive services.

Quasi-experimental studies on integrating family planning and immunization services have been conducted in Ghana, Rwanda, Togo, and Zambia. The studies in Rwanda and Togo found a significant increase in contraceptive use with no change in use of immunization services after services were integrated.^7^^,^^8^ On the other hand, in Ghana and Zambia, there was no statistically significant increase in contraceptive uptake, and immunization data were not monitored. However, process findings from Ghana and Zambia indicated that the model was not implemented as designed. In Zambia, family planning information was often given during group talks instead of during one-on-one counseling sessions, and in Ghana, family planning messages were not consistently communicated.^9^

Documented evidence and program learning around integrating immunization and family planning services remain fairly limited. Additional evidence is needed specifically on the effects of integrating family planning and immunization services on immunization coverage, especially in light of the extended negative impact on immunization programs in Cameroon, Nigeria, the Philippines, and elsewhere from past allegations by community or religious sectors that immunization was a disguised attempt to sterilize populations.^10^^–^^13^ Because of these gaps and concerns, the immunization community has expressed reservation about family planning and immunization integration until there is a robust evidence base indicating that this practice is not detrimental to achieving immunization goals. This paper describes one experience from Liberia that contributes to building that evidence base.

Starting in 2011, the Maternal and Child Health Integrated Program (MCHIP) began collaborating with the Liberian Ministry of Health and Social Welfare (MOHSW) to launch a proof-of-concept initiative to integrate family planning and routine immunization services at fixed health facilities. The MOHSW recognized the significant role that family planning could play in reducing maternal mortality in the country.

The 2007 Liberia Demographic and Health Survey (DHS) was the most recent source of population-based data for family planning and immunization available at the time of program design. It revealed a low modern contraceptive prevalence of 10.3% for married women of reproductive age^14^ (later increasing to 19.1% in the 2013 DHS^15^), and a special analysis of the 2007 DHS data showed short interpregnancy intervals (41% of pregnancies occurred within intervals shorter than 24 months).^16^ In addition, women in Liberia who were within 2 years postpartum experienced high unmet need for family planning (82%).^16^ Among women in this extended postpartum period, only 7% used any method of family planning, even though only 9% of women desired another birth within 2 years.^16^

The 2007 DHS data also indicated that among children aged 12–23 months, 75% received a first dose of DTP and 50% received the recommended 3 doses of DTP.^14^ (Receipt of the first and third dose of DTP increased to 91% and 71%, respectively, in the 2013 DHS.^15^) Liberia's national immunization schedule recommends infant vaccination visits at birth, at 6, 10, and 14 weeks, and at 9 months, with immunization to be offered daily at static health facilities and through outreach programs. However, sessions do not necessarily offer all vaccines on a given day, and the ages at which these visits actually take place may be somewhat later.

With the government's commitment to reduce high levels of unmet need for family planning and maternal mortality, senior staff at the MOHSW supported the concept of using routine immunization contacts to increase access to family planning. They also highlighted the need to maximize limited human and financial resources to achieve as broad a health benefit as possible.

The purpose of this article is to present the results of 9 months of implementation of a contextualized model for integrating family planning and EPI in Liberia as a means of increasing contraceptive use among postpartum women. The article also describes factors that enabled or hindered integrated service delivery and presents implications for integrating these services in other settings.

## PROGRAM DESCRIPTION

From March through November 2012, Liberia's MOHSW, with technical support from MCHIP and the United States Agency for International Development (USAID), piloted a model for integrating immunization and family planning services in 10 health facilities in Bong and Lofa counties. As a result of discussions with the MOHSW, the pilot was not designed with a rigorous research design; rather, it was intended to be a proof of concept to generate learning that would inform creation of a scalable model for implementation. The MOHSW's EPI, Family Health, and Health Promotion Divisions were closely involved in providing input on the design of the model and in selecting intervention facilities. Key steps in planning, implementing, and evaluating the project are highlighted in Table 1.

**Table 1.** Time Frame and Key Activities for the Integrated EPI-Family Planning Pilot Initiative in Liberia

**Time Frame**	**Activity**	**Remarks**
Feb 2011	Initial discussions with the MOHSW; stakeholder meeting with national EPI and family planning officials, county health teams, partners	Decision to work in Bong and Lofa; consensus to work only on facility-based integration for routine immunization
Apr–May 2011	Formative research to inform details of integration model	Sensitivity and stigma regarding postpartum women's use of family planning services revealed
Jun–Sep 2011	Design, pretesting, and production of training materials	Addresses perceptions regarding use of contraceptives by postpartum women noted during formative assessment
Feb 2012	Training of staff at 5 facilities each in Bong and Lofa	3-day training for vaccinators and family planning providers, including field practice; 1-day orientation for county supervisors and officers in charge
Mar–Nov 2012	Pilot of integrated EPI-family planning service delivery, including monthly supervision visits to participating facilities	Supervision by MCHIP staff accompanied by representatives from Family Health Division, EPI, and county health teams
Jul–Aug 2012	Refresher training and midterm assessment using quantitative and qualitative methods	Based on facility staff feedback, introduced privacy screens in vaccination area to enhance confidentiality of the mother's family planning decision
Dec 2012	Final assessment using quantitative and qualitative methods	Included focus group discussions with referral acceptors and non-acceptors, interviews with service providers and facility officers in charge, and interviews with partner agency representatives and supervisors
Mar 2013	Final stakeholder meeting	Presentation of approach and key findings to the MOHSW, partners, and county health teams from 6 counties

Abbreviations: EPI, Expanded Programme on Immunization; MCHIP, Maternal and Child Health Integrated Program; MOHSW, Ministry of Health and Social Welfare.

### Site Selection

The proof of concept was conducted at 10 health facilities in Bong and Lofa counties; these counties were selected due to their relatively strong EPI performance. Contraceptive prevalence in the regions where these counties are located fell below the national rate.^14^ One hospital and 4 clinics were purposively selected in each county with guidance from the MOHSW. Fixed facilities were chosen instead of outreach services because a greater proportion of children are vaccinated at fixed facilities than through outreach services. In addition, fixed services tend to be more reliable and offer more privacy.

Pilot facilities had relatively strong immunization performance, but contraceptive use in the pilot regions was low.

### Formative Assessment Findings

In 2011, we conducted a formative assessment in 4 of the intervention health facilities to inform development of the integration model, messages, and communication materials. The assessment consisted of focus group discussions (FGDs) with mothers of infants under 1 year of age and interviews with vaccinators, family planning providers, and health facility officers in charge.

The assessment revealed that stigma about returning to sexual activity and using family planning before the baby walks or turns 1 year of age acted as a major barrier to using postpartum family planning services. Many respondents also believed that premature return to sexual activity and contraceptive use could “spoil” the breast milk and harm the baby. Family planning providers and vaccinators highlighted the importance of privacy and one-on-one communication as factors that could affect women's willingness to seek family planning services. The assessment also revealed that women were not routinely referred from immunization to family planning services and that women rarely sought family planning services during immunization visits. Vaccinators, family planning providers, and clients all expressed support for the idea of linking family planning and immunization services.

Stigma about contraceptive use before the baby walks or turns 1 year of age prevented many women from using postpartum family planning.

### Intervention Design

**Figure 1.**
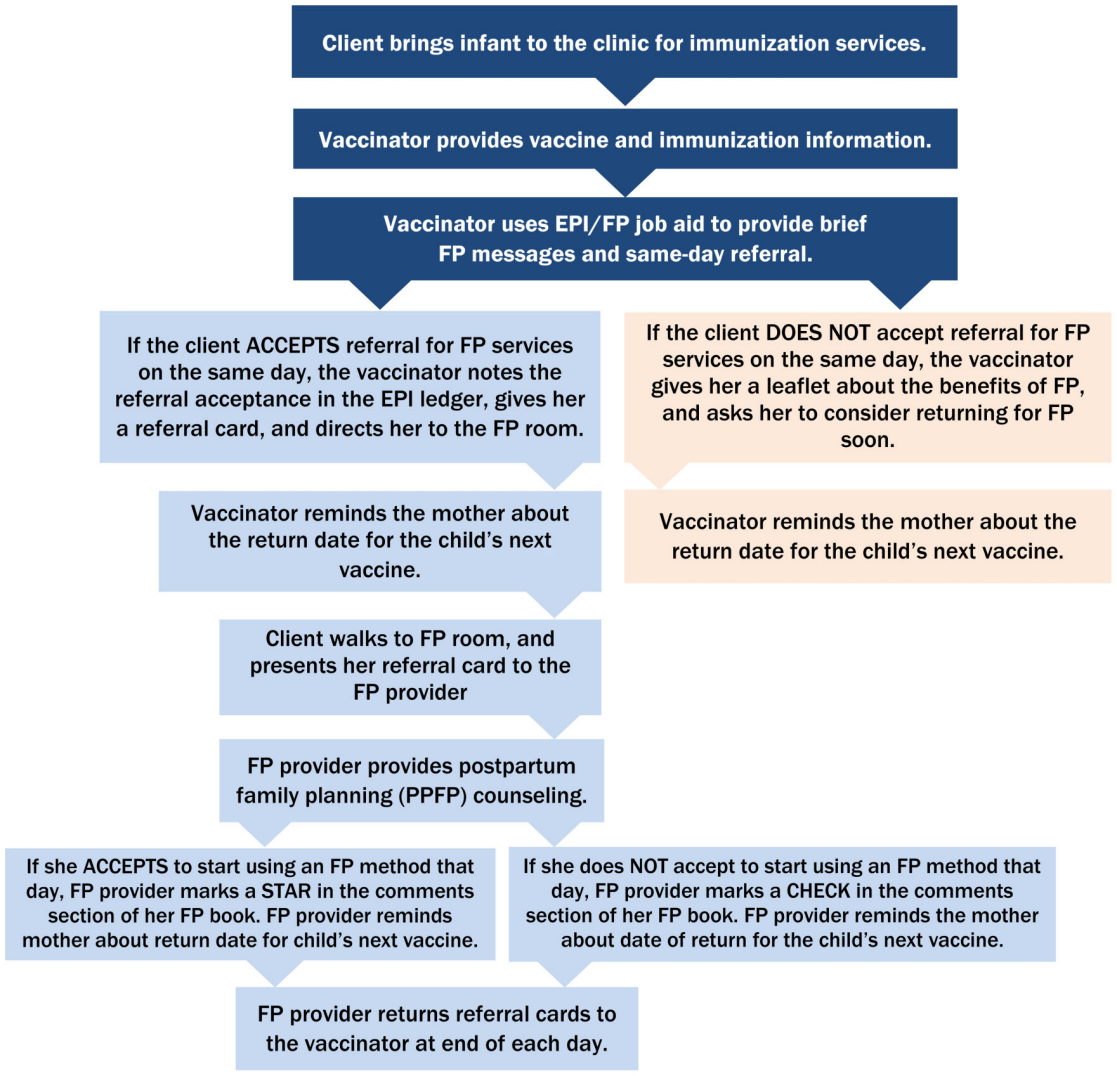
Integrated EPI-Family Planning Service Delivery Model in Liberia Abbreviations: EPI, Expanded Programme on Immunization; FP, family planning.

**Figure 2.**
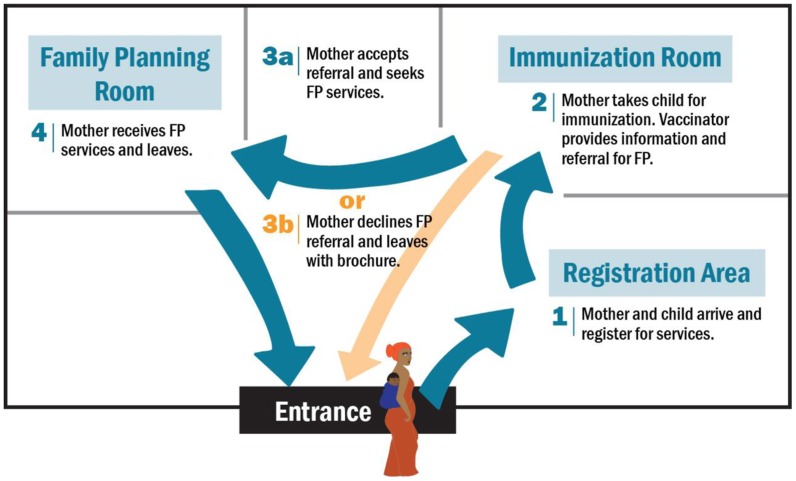
Client Flow for Integrated EPI-Family Planning Services Abbreviation: EPI, Expanded Programme on Immunization.

MCHIP, in consultation with the MOHSW, developed an integration model, which was informed by the formative assessment findings and experiences in other countries. The integrated model emphasized co-located provision of same-day, facility-based services: vaccinators were trained to provide brief, targeted family planning and immunization messages and same-day family planning referrals to mothers bringing their infants to the facility for routine immunizations. Specifically, at the completion of each vaccination contact, vaccinators were directed to use a simple job aid to share targeted messages one-on-one (not through group health talks) with each mother and to then offer her a referral to a co-located family planning room for more in-depth family planning counseling and services (Figure 1). The approach was designed to minimize the impact on the typical patient flow for immunization. The client flow within the health facility, from arrival at a facility to departure, is illustrated in Figure 2.

The integrated model emphasized co-located provision of same-day immunization and family planning services.

We developed strategically designed behavior change communication materials, including a job aid, poster, and brochure, to help standardize communication by vaccinators and reinforce key messages provided to mothers (see supplementary materials). The messages emphasized that family planning is safe for use by women with young babies and that it is acceptable for women to use family planning even before the baby walks. The job aid was designed to be simple and user-friendly, with clear step-by-step directions for vaccinators. The poster included a photo of a breastfeeding woman seeking family planning services, along with messages that “family planning is good for baby ma” and that encouraged women to “Go for family planning today!” The job aid guided vaccinators to reference the poster during the immunization contact. Women who declined to go for family planning services on the same day received a brochure as a take-home reference, highlighting information about the benefits of family planning for mother, father, baby, and for general family well-being. Clients were encouraged to share the brochure with their spouses, other family members, and friends in order to spark discussion about family planning.

After designing the integration model and preparing and pretesting the messages and materials, we trained at least 1 family planning provider and 1 vaccinator per health facility. The 3-day training covered concepts relevant to postpartum family planning and immunization and values clarification, oriented participants to the new integration approach and tools, and allowed participants to apply the skills in a service delivery setting. We also oriented health facility, district, and county supervisors to the approach.

### Monitoring and Supervision

During the 9-month implementation period, MCHIP staff and MOHSW representatives conducted monthly supervision visits to each site. Supervisors monitored service provision, collab-oratively reviewed and documented service statistics (including immunization and family planning service data and referrals made from immunization to family planning services), conducted exit interviews with clients, and provided feedback and developed action plans with each of the facility teams. A refresher training and midterm assessment were also conducted halfway through the implementation period.

The midterm assessment and ongoing supervision indicated that the model was generally being implemented as planned, but several challenges were uncovered. Most notably, a lack of privacy at the EPI station prevented some women from accepting family planning referrals, especially at facilities where vaccinations were given in a public space. Other challenges included human resource constraints, extended waiting times for family planning services, and vaccine and contraceptive commodity stock-outs.

Lack of privacy during immunization prevented some women from accepting family planning referrals.

In light of these and other observations, we made a number of important adjustments, including: encouraging teams to develop facility-specific plans for managing increased family planning client load; advocating improvements in the commodity supply chain; encouraging service providers (both vaccinators and family planning providers) to set weekly meetings in order to improve communication and coordination; and introducing privacy screens at facilities where vaccinations were conducted in public areas of the facility. These screens provided visual privacy for clients and a quieter space for child vaccination, and they reduced the likelihood of others watching or listening to clients' conversations with the vaccinator.

## FINAL ASSESSMENT METHODS

In December 2012, MCHIP and the MOHSW conducted a final assessment of the integrated approach, consisting of a review of service statistics as well as in-depth interviews and FGDs. The objectives of the assessment were to evaluate whether the pilot was associated with changes in family planning and immunization outcomes and to gather qualitative information on lessons learned. A protocol for conducting secondary analysis of data generated by the program was submitted to and was exempted from human subjects review by the Johns Hopkins Bloomberg School of Public Health's Institutional Review Board.

### Service Statistics

Family planning service data were gathered on the:

- Number of clients accepting family planning referrals from the vaccinators
- Number of EPI-referred clients who followed through on the family planning referrals
- Number of EPI-referred clients who accepted a contraceptive method the same day
- Total number of new contraceptive users at the intervention facilities (defined in this paper as both EPI-referred and other clients who either started using a contraceptive method or reinitiated use after childbirth)
- Contraceptive method mix among EPI referral acceptors

These data were obtained from family planning registers and supplemental “EPI-Family Planning registers” at participating facilities during monthly supportive supervision visits. The data were analyzed to compare service utilization at intervention sites during the pilot phase (March–November 2012) against the same months from the previous year (March–November 2011). It was not possible to compare family planning data from the pilot sites against other facilities in these counties, as county-level family planning data were not available from the MOHSW. Same-day referral acceptance was calculated by dividing the total number of referral acceptors by the total number of infants immunized (bacille Calmette-Guérin [BCG] + measles + Penta 1 + Penta 2 + Penta 3) in each facility during each month, as derived from vaccination registers.

Immunization indicators monitored included the number of Penta 1 and Penta 3 doses administered, as well as the dropout rate from the Penta 1 to Penta 3 doses administered. Penta 1, 2, and 3 refer to the first, second, and third doses of pentavalent vaccine, which contains antigens for diphtheria, pertussis, tetanus, hepatitis B, and *Haemophilus influenzae* type b. For this analysis, routine immunization administrative data provided by the MOHSW in February 2013 were used to compare the:

- Number of Penta 1 and Penta 3 doses administered at pilot sites from March–November 2012 with those administered at pilot sites during the same period in 2011
- Number of Penta 1 and Penta 3 doses administered at pilot facilities in the 2012 calendar year with those administered at all other facilities in Bong and Lofa counties in the same year
- Penta 1 to Penta 3 dropout rates at the pilot facilities in Bong and Lofa in 2012 with the dropout rates at all other facilities in each respective county in the same year.

### Qualitative Data

We conducted in-depth interviews with purposively selected personnel: service providers (vaccinators and family planning providers), officers in charge, program managers, partner agency representatives, and supervisors (MCHIP, district, county, and MOHSW representatives). We also conducted FGDs with clients of participating health facilities—both those who had and those who had not accepted the family planning referrals from the vaccinators. Health facilities were randomly assigned to either recruit referral acceptors or non-acceptors for the FGDs. During the period preceding the assessment, at each facility selected to recruit family planning referral acceptors, vaccinators were directed to invite all women who accepted the family planning referral to participate in the FGDs until they had recruited 7–10 participants. At facilities assigned to conduct FGDs with referral non-acceptors, vaccinators were directed to invite every third mother who did not accept the family planning referral until they had recruited 7–10 participants. All interview and FGD participants provided oral consent to participate. Table 2 describes the number and characteristics of respondents in the final assessment.

**Table 2.** Composition of Key Informant Interviews and Focus Group Discussions

**Description of Participants**	**No. of Participants**
**Key Informant Interviews**	**42**
Vaccinators	10
Family planning providers	10
Officers in charge	9
Program managers, partner agency representatives, supervisors	13
**Focus Group Discussions (FGDs)^a^**	**56**
4 FGDs with family planning referral acceptors	31
4 FGDs with non-acceptors	25

a Health facility staff were not present during FGDs to minimize their potential influence on client responses.

### Data Analysis

Quantitative data were entered into Microsoft Excel. Qualitative data were transcribed from pre-formulated questionnaires into an electronic database. We then prepared simple frequencies and trend analyses from the quantitative service data and conducted a thematic analysis from the qualitative data.

## RESULTS

### Family Planning Referral Acceptance and Completion

Service data revealed wide variation in family planning referral acceptance across pilot facilities. On average, the percentage of mothers bringing their children for immunization who accepted a family planning referral on the same day ranged from 10% to 45% per month across facilities in the 2 counties (Table 3). There was no clear trend in referral acceptance over time, across facilities, or between the 2 counties; however, hospitals generally had lower percentages of clients accepting referrals than clinics.

**Table 3.** Family Planning (FP) Referrals and Use at Pilot Sites During Intervention Period (March–November 2012), by County

**Facility**	**Average Monthly % of Mothers Accepting FP Referrals From Vaccinators**	**Total No. of Mothers Accepting FP Referrals**	**No. (%) of Referral Acceptors Who Went to the FP Provider**	**No. (%) of Referral Acceptors Who Went to the FP Provider and Accepted a Method That Day**
**Bong County**		**1,064**	**934 (88%)**	**892 (96%)**
Fenutoli Clinic	26.0%	99	85 (86%)	80 (94%)
Garmu Clinic	45.2%	361	342 (95%)	328 (96%)
Zoweinta Clinic	15.7%	159	138 (87%)	126 (91%)
Salala Clinic	9.9%	241	191 (79%)	186 (97%)
Phebe Hospital	12.5%	204	178 (87%)	172 (97%)
**Lofa County**		**426**	**357 (84%)**	**332 (93%)**
Borkeza Clinic	12.4%	51	43 (84%)	40 (93%)
Ganglota Clinic	34.1%	86	54 (63%)	53 (98%)
Gbonyea Clinic	32.4%	80	72 (90%)	71 (99%)
Kpaiyea Clinic	24.3%	65	54 (83%)	52 (96%)
Curran Hospital	14.4%	144	134 (93%)	116 (87%)

During the 9-month pilot period, a total of 1,490 mothers accepted a family planning referral from a vaccinator (426 in Lofa county, 1,064 in Bong county). Of mothers who accepted a family planning referral, 84% and 88% completed the same-day referral in Lofa and Bong, respectively. Among those women who completed the referral, 93% and 96% in Lofa and Bong, respectively, accepted a contraceptive method that day.

Over 80% of mothers who accepted a family planning referral completed the referral, and, in turn, over 90% of these women accepted a method that day.

Adding privacy screens to immunization areas helped motivate family planning referral acceptance and follow-through. The screens enabled clients to focus more on the information shared by the vaccinator and helped clients to avoid feeling stigmatized when accepting the family planning referral.

The main reasons for not accepting a family planning referral cited by clients included long wait times to see a family planning provider, unclear pathways from the vaccination station to the family planning room, and lack of privacy (Table 4). For women who accepted a referral but did not accept a contraceptive method that day, common reasons included wanting to discuss the decision with their partner first, wanting to wait until the baby was older, and dissuasion by the family planning provider from using a contraceptive method before reaching a particular time postpartum (for example, waiting until 6 weeks after childbirth). All respondents (clients, service providers, supervisors, and partner organizations) expressed a desire for the integrated service delivery approach to continue.

**Table 4.** Factors Enhancing Implementation of the Integrated EPI-Family Planning Model: Results of In-Depth Interviews and Focus Group Discussions^a^

**Category**	**Factor**
Infrastructure	• Proximity of family planning and immunization services to each other and clarity of pathways between service sites
	• Privacy for clients (at immunization stations in particular)
Management, staffing, and coordination	• Availability of vaccinators and family planning providers on the same day
	• Frequent communication between vaccinators and family planning providers
Training and supportive supervision	• Regular supportive supervision
	• On-the-job training for new staff
Supplies	• Reliable commodity supply (vaccines and contraceptives)
Behavior change communication	• Job aids or reminder materials to reinforce key steps of the referral process
	• Good-quality counseling

Abbreviation: EPI, Expanded Programme on Immunization.

a With clients, service providers, supervisors, and partner organizations.

### Trends in Family Planning Use

The total number of new contraceptive users at participating facilities (i.e., among EPI-referred clients who accepted a method the same day as well as other clients) increased by 90% in Lofa (517 to 983) and by 73% in Bong (1,182 to 2,039) between March–November 2011 and March–November 2012. It should be noted that during the pilot period (March–November 2012), the number of new contraceptive users in pilot sites included women who committed to using the lactational amenorrhea method (LAM). In 2011, LAM was not routinely tracked in family planning registers, but routine counseling on LAM by family planning providers and active use of LAM were suspected to be very low prior to the pilot.

Women who were referred from EPI and who accepted a contraceptive method on the same day accounted for a substantial proportion of the total new contraceptive users at the pilot sites. In participating facilities in Bong and Lofa, 44% and 34%, respectively, of the total number of new contraceptive users were EPI-referred. No other major efforts to increase family planning uptake took place during the pilot phase of the integration initiative, except for 1 facility in Lofa county, which began providing community-based family planning during this time.

**Figure 3.**
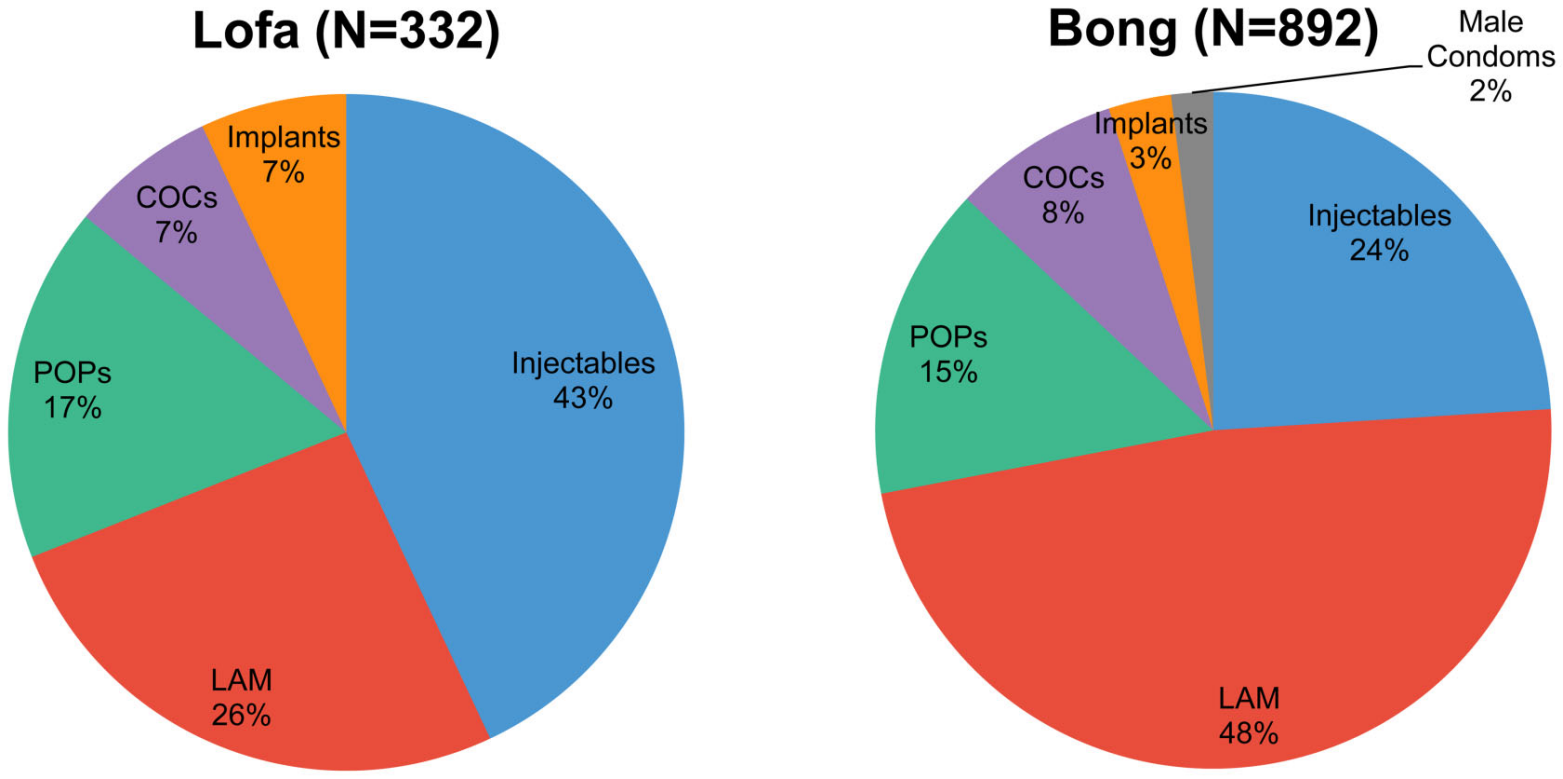
Contraceptive Method Mix Among Same-Day Referral Acceptors in Pilot Facilities, Lofa and Bong Counties, March–November 2012 Abbreviations: COCs, combined oral contraceptive pills; LAM, lactational amenorrhea method; POPs, progestin-only pills.

Among EPI-referred women who accepted a contraceptive method on the same day, the method mix varied between the counties. In Bong, nearly half of the same-day referred family planning acceptors committed to using LAM, while about one-quarter chose injectables and nearly one-quarter chose oral contraceptive pills (Figure 3). In Lofa, most same-day referral family planning acceptors (43%) chose injectables, while about one-quarter chose LAM and another quarter chose pills. Less than 10% of women chose implants in either county. Although intrauterine devices (IUDs) were offered in most facilities in Liberia, none of the family planning referral acceptors were provided an IUD on the same day. It should be noted that the method mix captured in this figure represents only EPI-referred women, not all contraceptive users. Method mix for all new contraceptive users beyond the same-day referral acceptors was not tracked.

**Figure:**
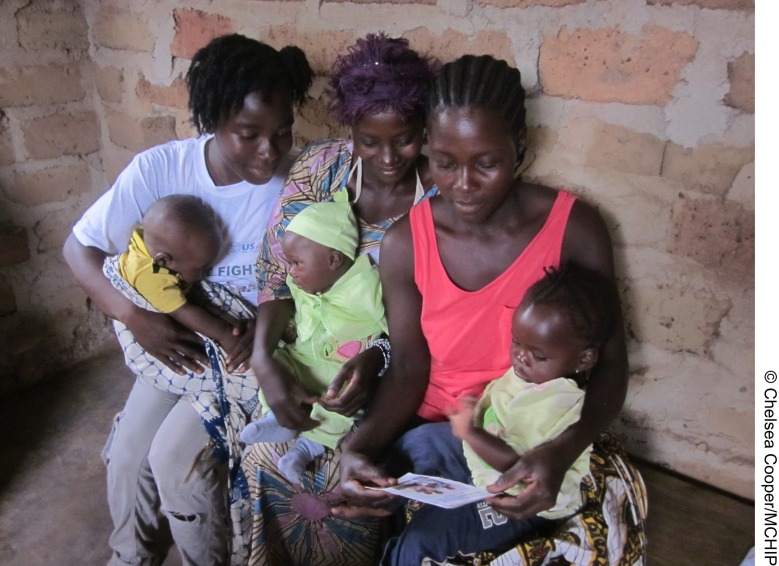
Mothers bringing their children for vaccination review a family planning brochure provided by the vaccinator.

LAM was the most popular method among immunization-referral acceptors in Bong, while injectables were the most popular in Lofa.

Results from FGDs indicated that the reach of this intervention was amplified beyond vaccinators' one-on-one communication with clients: many mothers reported sharing the information and take-home materials about family planning with friends, family members, and partners. One client said, “My friend said that her baby was small, and I talked to her about family planning. I told her to come on her rightful time for vaccine and she can get family planning.” The qualitative data also revealed that the integrated services improved knowledge and changed views about family planning among clients and providers alike. For example, several clients and family planning providers reported that before the intervention, they did not know that a woman with a young infant could use contraception.

### Trends in Use of Immunization Services

Immunization data revealed an increase in the number of Penta 1 and Penta 3 doses administered across pilot sites during the implementation period compared with the same period of the previous year. In Lofa, pilot facilities administered 35% more Penta 1 doses and 21% more Penta 3 doses from March–November 2011 to March–November 2012 (Table 5). In contrast, the data for non-pilot facilities showed decreases in Penta 1 (11%) and Penta 3 (6%) administration during the same time period. In Bong, there was a modest increase in Penta 1 (9%) and Penta 3 (5%) doses given at pilot facilities from 2011 to 2012. However, these increases were smaller than those experienced in all other facilities in Bong county.

**Table 5.** Number of Penta 1 and Penta 3 Doses Administered at Pilot and Non-Pilot Facilities by County, Pre-Intervention Period (March–November 2011) vs. Intervention Period (March–November 2012)

****	**Lofa County**						**Bong County**					
****	**Pilot Facilities (N = 5)**			**All Other Facilities (N = 48)**			**Pilot Facilities (N = 5)**			**All Other Facilities (N = 31)**		
****	**Before**	**During**	**% Change**	**Before**	**During**	**% Change**	**Before**	**During**	**% Change**	**Before**	**During**	**% Change**
Penta 1 doses	533	721	+35%	8,095	7,244	−11%	2,303	2,508	+9%	8,673	9,583	+10%
Penta 3 doses	458	553	+21%	7,456	7,012	−6%	2,175	2,280	+5%	8,063	8,926	+11%
Penta 1 to Penta 3 dropout rate	14%	25%		8%	3%		6%	9%		7%	7%	

In both counties, the increase in Penta 1 was more than that of Penta 3 at the pilot facilities. The smaller increase in Penta 3 resulted in a net increase in the Penta 1 to Penta 3 dropout rate (a standard parameter in immunization program management and evaluation that is based on a simple ratio of Penta 3 to Penta 1 doses administered). In Lofa county, the Penta 1 to Penta 3 dropout rate increased from 14% in 2011 to 25% in 2012 at pilot facilities, while it decreased from 8% to 3% in all other facilities. In Bong county, the Penta 1 to Penta 3 dropout rate increased from 6% to 9% at pilot facilities, while it remained unchanged at 7% in all other facilities. The dropout rate in pilot facilities in Bong was still below the 10% threshold designated by the World Health Organization (WHO) as indicating a problem with immunization dropout.^17^

Penta 1 and Penta 3 administration increased across the pilot sites, but more so for Penta 1, resulting in a net increase in the Penta 1 to Penta 3 dropout rate.

Further examination revealed that pilot facilities in Lofa had higher dropout rates than non-participating facilities even prior to participating in the intervention. In Bong, findings from the pilot facilities were disproportionately affected by data from 1 large facility that experienced a drop-off in immunization performance from 2011 to 2012, which was attributed to human resource constraints and internal supervisory turnover during the intervention period.

In qualitative interviews, some vaccinators reported greater confidence in their roles and perceived that their value within the health system and community had increased as a result of the intervention. In addition, interviews with service providers (vaccinators, in particular) suggested that the intervention may have contributed to greater staff appreciation for recordkeeping. None of the clients participating in the FGDs (neither referral acceptors nor non-acceptors) reported feeling discouraged to return to the facility for vaccinations. Rather, clients reported that they saw the value of vaccinating their child and would return regardless of their decision to accept a family planning referral.

## DISCUSSION

The experience and results of this pilot project contribute to the global evidence base on the integration of 2 lifesaving services—immunization and family planning. In our proof of concept, we found that a simple model of counseling and referrals from immunization services to same-day, co-located family planning services increased postpartum contraceptive uptake in 2 counties of Liberia and, at a minimum, did not decrease vaccination administration.

### Impact on Family Planning

It is likely that some of the women who accepted family planning referrals from the vaccinator may have come to use family planning anyway eventually, but given the high rate of short interpregnancy intervals in Liberia, any earlier uptake of postpartum family planning is also beneficial. Several women indicated during the FGDs that they had felt motivated by the family planning information shared by the vaccinator, but they had needed to speak with their husbands before making a decision and had returned for a contraceptive method at a later date. (These women would not have been captured as same day-referred family planning acceptors but could have contributed to overall increases in new contraceptive users.) Clients reported amplifying family planning messages provided by the vaccinators with other family members and peers.

In both pilot counties, there was high acceptance of LAM among same-day referral acceptors. LAM is a highly effective contraceptive method when practiced correctly, and it also has great benefits for infant health. In addition, there is evidence that LAM is a gateway method to other modern contraceptives even though LAM is a temporary method.^18^ However, it is important that health workers communicate effectively about LAM to ensure clients understand how to use the method correctly and understand the importance of follow-up to promote timely transition from LAM to another modern method.

The monthly average percentage of mothers bringing their children for immunization who accepted a family planning referral on the same day may underrepresent the actual same-day referral acceptance, as the denominator used was the total number of infants immunized (BCG + measles + Penta 1 + Penta 2 + Penta 3) in each facility each month, as derived from vaccination registers. For the purpose of these calculations, BCG, measles, Penta 1, Penta 2, and Penta 3 vaccinations were all treated as distinct contacts; however, in reality, infants may receive more than one of these vaccines during a contact with EPI services. Furthermore, a woman who accepts a contraceptive method at one visit and later returns for subsequent immunization visits is less likely to need or accept a family planning referral at those later visits.

### Factors Contributing to Successful Family Planning Referrals

Routine use of a simple, strategically designed job aid was instrumental in supporting vaccinators to communicate intended family planning messages to clients. A formative assessment conducted to inform project design allowed for the issue of stigma around contraceptive use to surface and to be addressed in the design of the integration model and its communication materials. Improving client privacy at the vaccination stations also enabled women to more freely accept family planning referrals. The provider training contributed to changes in provider knowledge and attitudes about the acceptability and effectiveness of offering family planning services to women with infants, which seemed to improve subsequent provider practices.

### Impact on Immunization

The immunization findings pose some challenge to interpretation. Administration of both Penta 1 and Penta 3 increased at the pilot sites in both counties relative to the same period for the previous year, albeit less so for Penta 3. In Bong, this increase was somewhat lower in pilot facilities than in all other facilities. The increased dropout rate at pilot facilities in Bong was still below the threshold that WHO considers representing a problem.^17^ In Lofa, by contrast, an increase in the dropout rate was observed at pilot facilities whereas it fell at all other facilities. However, pilot facilities in Lofa experienced a dramatic increase of 35% and 21% in doses of Penta 1 and Penta 3 administered, respectively, whereas figures for Penta 1 and Penta 3 actually decreased in non-pilot facilities. That is, the pilot facilities experienced a substantial net gain in doses administered despite the increased dropout rate.

Follow-up visits to pilot facilities pointed to an unusual service delivery system in the Lofa subdistrict in which all 5 pilot facilities were located: Penta 1 doses were provided by each clinic whereas Penta 3 and other later doses in the vaccination schedule were administered mostly by an outreach team fielded by a nearby private hospital. This situation highlights the need to thoroughly understand the health system context at the micro level, particularly in the case of small-scale pilot activities.

Nevertheless, the increase in the dropout rates observed at pilot facilities in both counties underscores the need for vaccinators and family planning providers alike to inform mothers of the need to return for their child's next vaccination and the importance of fully completing the immunization series. Additional experience and learning is also needed on the development and use of robust data collection systems for integrated services, the types of changes to anticipate and monitor, and attribution of changes to the integration process itself, in particular to ensure that integrating family planning with existing immunization services does not have a negative impact on immunization performance.

It is critical for both vaccinators and family planning providers to inform mothers of when they need to return for their child's next vaccination.

### Scaling-Up the Integrated Model

Previous experience with integration of family planning and immunization services in other countries revealed a need for systems that are clear and user-friendly and that pose minimal added burden to vaccinators.^9^ While our service delivery model is fairly simple, we did provide significant program support, including formative assessment, training for both immunization and family planning providers, job aids, privacy screens, and monthly supervision. The stakeholders involved in this pilot viewed the proof-of-concept phase as an opportunity to refine an effective integration model; as such, more substantial inputs arguably were required during this phase (e.g., formative research, pretesting of messages and materials, midterm assessment, client exit interviews) than would be necessary on an ongoing basis within an expanded approach. At the same time, scale-up is well known to be a difficult process requiring additional inputs and adaptation. Using the proof-of-concept experience, MCHIP has developed an implementation package outlining the process, training materials, tools, and key implementation considerations to help government and other stakeholders adapt and scale-up the approach (see supplementary material).

Return visits to the implementation sites conducted several months after the pilot period revealed that the EPI-family planning referral process was still in place. District and county teams had been closely involved in the rollout of the activity and were committed to continuing the approach at the focus sites beyond the proof-of-concept phase. In addition, from the earliest stages of this activity, the MOHSW, county teams, and donors expressed interest in scaling-up the integrated approach to additional sites, should the proof of concept be successful. In light of the final assessment findings, the MOHSW officially endorsed the approach for scale-up within additional counties, with the caveat of incorporating additional mechanisms for reinforcing immunization services. Adjustments will be incorporated as the integrated model is expanded, in particular, to address the issue of immunization dropout—a known challenge in the Liberian context even in areas with strong overall EPI performance. These include taking appropriate measures to assure privacy during EPI and family planning service provision; ensuring that there is a clear pathway from one service to the other; and improving client-provider communication (for example, ensuring that family planning messages are given at every vaccination contact, and that vaccinators and family planning providers remind mothers to return for their child's next immunization visit).

Future recommendations include strengthening family planning and immunization commodity security and improving recordkeeping practices, such as recording acceptance or refusal of the family planning referral directly in the EPI register. The MOHSW also recommended that take-home communication materials with immunization messages should be provided to women during service contacts to reinforce key immunization information.

Findings from the 2013 Liberia DHS indicate that 71% of children 12–23 months old received 3 doses of pentavalent vaccine,^15^ a marked increase from the 2007 figure of 50%.^14^ This increase in immunization coverage offers a more favorable environment and stronger platform for reaching women with other needed services. Certainly, continued attention to further increasing immunization coverage, improving quality of care, enhancing data collection, and reducing dropout will be critical for further strengthening these services.

### Limitations

This project has important limitations, including its small sample size (in terms of the facilities and individual respondents). In addition, the assessments were conducted by MOHSW and MCHIP representatives, not an external evaluator. The makeup of the final assessment team (MCHIP staff and MOHSW representatives who were familiar with staff at the facilities) could have influenced the feedback provided by some respondents. Challenges were encountered with data quality and availability during the final assessment, resulting in an inability to draw comparisons in family planning indicators between intervention and non-intervention sites in the 2 pilot counties. Finally, the assessment design did not allow obtaining data on family planning continuation rates or the incidence of closely spaced births.

## CONCLUSION

In this proof of concept, integrating immunization and family planning services, using a referral model with co-located services designed for Liberia's health system and sociocultural environment, was feasible, resulting in increased contraceptive uptake among postpartum women. Immunization-related findings are encouraging but less clear, indicating that there was at least no decrease in the number of vaccination doses administered in conjunction with the integrated model. While continuous monitoring of immunization outcomes is needed, scaling-up this model could potentially contribute to large increases in postpartum contraceptive uptake, leading to longer birth intervals and, ultimately, to improved health outcomes for children and mothers and to other socioeconomic benefits for families.

## Notes

**Peer Reviewed**
